# Metabolic Dysfunction and Related Dietary Exposures in Relation to Pancreatic Cancer Risk: A Review of the Evidence

**DOI:** 10.3390/ijms27177707

**Published:** 2026-08-28

**Authors:** Junsu Yang, Yicong Huang, Artitaya Lophatananon, Kenneth R. Muir

**Affiliations:** Division of Population Health, Health Services Research and Primary Care, School of Health Sciences, Faculty of Biology, Medicine and Health, The University of Manchester, Manchester M13 9PT, UK; junsu.yang@postgrad.manchester.ac.uk (J.Y.); yicong.huang@manchester.ac.uk (Y.H.); artitaya.lophatananon@manchester.ac.uk (A.L.)

**Keywords:** pancreatic cancer, metabolic risk factor, diet risk factor, review

## Abstract

Pancreatic cancer is a highly lethal malignancy with an increasing global incidence. Although metabolic dysfunction has been implicated in pancreatic cancer risk, the strength and consistency of evidence across different metabolic traits remain uncertain. Evidence for dietary exposures related to metabolic regulation is also less clearly established. This umbrella review synthesised evidence from systematic reviews, meta-analyses, and umbrella reviews evaluating metabolic dysfunction and related dietary exposures in relation to pancreatic cancer risk. A systematic search of PubMed, Web of Science, and PROSPERO identified reviews published between 1 January 2021 and 1 February 2026. Seventeen reviews met the inclusion criteria. The primary search focused on metabolic dysfunction. Dietary exposures identified during the screening process were retained as a secondary evidence domain when they were considered relevant to glycaemic regulation, insulin response, adiposity, or wider metabolic health. Primary-study overlap was slight, with an overall corrected covered area (CCA) of 1.59% and weighted corrected covered area (wCCA) of 1.40%; within the principal evidence domains of adiposity-related exposures and metabolic dysfunction, the CCA and wCCA were 1.15% and 1.81%, respectively. Metabolic dysfunction demonstrated the strongest and most consistent associations with pancreatic cancer risk. Metabolic syndrome, diabetes mellitus, hyperglycaemia, and abdominal obesity were graded as probable evidence. Hyperglycaemia showed the strongest component-level association (RR = 1.55, 95% CI: 1.42–1.70), while diabetes mellitus was consistently associated with increased risk across both cohort and case–control studies. Abdominal obesity, assessed using waist circumference and waist-to-hip ratio, was also positively associated with risk. General obesity, prediabetes, and reduced HDL cholesterol were graded as limited/suggestive evidence, whereas hypertriglyceridaemia was graded as limited/no conclusion. Related dietary exposures generally showed weaker and less consistent associations, with no exposure supported by convincing evidence. Overall, pancreatic cancer risk was more consistently associated with systemic metabolic dysfunction, particularly impaired glucose regulation, diabetes, metabolic syndrome, and abdominal adiposity, than with the related dietary exposures examined. However, the evidence remains predominantly observational and does not establish causality.

## 1. Introduction

The pancreas is a multifunctional organ located in the abdomen with both exocrine and endocrine roles. Its exocrine component contributes to digestion through the secretion of pancreatic enzymes, while its endocrine component regulates glucose homeostasis via insulin, glucagon and other hormones [1]. Malignant transformation of pancreatic tissue results in pancreatic cancer, a highly aggressive malignancy characterised by rapid progression and poor prognosis [2,3].

Pancreatic cancer remains one of the most lethal malignancies worldwide [3,4]. The dismal survival outcomes are largely attributable to late-stage diagnosis and the limited proportion of patients eligible for potentially curative surgical resection. Fewer than 20% of patients present with resectable disease at diagnosis, as many cases are already locally advanced or metastatic [2,3]. Even with advances in systemic therapies and improvements in diagnostic imaging, overall prognosis remains extremely poor [3,4].

Given these challenges, identifying modifiable risk factors and high-risk populations is essential for improving prevention and early detection strategies.

### Epidemiology

Pancreatic cancer ranks among the leading causes of cancer-related death globally and predominantly affects older adults, with incidence increasing markedly after the age of 60 years [3,4]. According to GLOBOCAN 2022 estimates, pancreatic cancer represented approximately 2.6% of incident cancers but accounted for around 4.8% of cancer deaths worldwide, highlighting its high mortality rate among all categories of cancer [4].

The global burden of pancreatic cancer has increased substantially over recent decades. Data from the Global Burden of Disease Study 2021 indicate that the number of new cases rose from approximately 207,905 cases to about 508,532 cases over the past two decades [5]. This upward trend has been attributed in part to population ageing and demographic changes [3,5]. Recent projections from the Global Cancer Observatory further indicate that this upward trend is expected to continue in the coming decades. According to GLOBOCAN 2025 estimates, the global number of new pancreatic cancer cases and deaths is projected to increase steadily from 2022 to 2050 for both males and females, largely driven by population ageing and demographic expansion [2,6]. Furthermore, the mortality curves for both genders closely parallel incidence curves, underscoring the persistently poor prognosis associated with this malignancy. These projections highlight the urgent need for improved prevention strategies and early risk identification.

In addition to demographic factors, several established risk factors contribute to pancreatic carcinogenesis. Cigarette smoking, chronic pancreatitis, and genetic predisposition have been consistently associated with increased risk [2]. Increasing attention has also been directed towards metabolic disorders. The global rise in obesity and type 2 diabetes parallels the increasing burden of pancreatic cancer in many regions [3,5]. The evidence base has evolved from the assessment of isolated anthropometric and dietary exposures towards a more integrated evaluation of metabolic dysfunction. Earlier studies and evidence syntheses primarily focused on general obesity, body mass index, diabetes, and individual dietary components. More recent reviews have examined abdominal adiposity, prediabetes, the timing of diabetes onset, metabolic syndrome components, and Mendelian randomization evidence. This development indicates a shift in focus from isolated exposure factors to interconnected metabolic risk profiles.

Previous evidence syntheses have generally evaluated obesity, diabetes, metabolic syndrome, or individual dietary exposures separately. Therefore, current evidence remains inconclusive as to whether the various exposure factors exert independent effects or whether there exists a broader pattern of interconnected metabolic dysfunction. Differences in methodological quality, overlap among primary studies, variations in exposure definitions, and the temporal relationship between diabetes and pancreatic cancer complicate comparisons across reviews.

This umbrella review was primarily designed to evaluate metabolic dysfunction in relation to pancreatic cancer risk, including metabolic syndrome, general and abdominal adiposity, diabetes mellitus, prediabetes, hyperglycaemia, insulin resistance, and dyslipidaemia. During the initial screening process, several dietary exposures related to metabolic regulation were also identified and were therefore evaluated as a secondary evidence domain.

The objectives were to (1) synthesise recent evidence on metabolic dysfunction and related dietary exposures in relation to pancreatic cancer risk; (2) compare the methodological quality and strength of evidence across exposure domains; and (3) develop an interpretative framework linking upstream dietary and adiposity-related exposures with systemic metabolic dysfunction.

## 2. Methods

This review was conducted in accordance with the Preferred Reporting Items for Systematic Reviews and Meta-Analyses (PRISMA) 2020 guidelines [7].

### 2.1. Search Strategy

The review was initially designed to evaluate metabolic dysfunction and related metabolic risk factors for pancreatic cancer. The primary search therefore focused on metabolic syndrome, obesity, body mass index, diabetes mellitus, hyperglycaemia, fasting glucose, insulin resistance, and related metabolic traits.

The literature search was conducted in PubMed, Web of Science, and the PROSPERO registry using Boolean operators, controlled vocabulary where applicable, and free-text keywords. Pancreatic cancer-related terms were combined with metabolic exposure terms and terms identifying systematic reviews, meta-analyses, or umbrella reviews.

In PubMed, the search combined pancreatic cancer-related terms with metabolic exposure terms and was restricted to systematic reviews using publication-type filters. The search syntax incorporated terms such as “pancreatic cancer,” “pancreatic ductal adenocarcinoma (PDAC),” “metabolic syndrome,” “hyperglycaemia,” “insulin resistance,” “type 2 diabetes,” “obesity,” and “body mass index,” combined with a systematic review filter. The searching code was written as (“pancreatic cancer”[Title/Abstract] OR “pancreatic neoplasm*”[Title/Abstract] OR “pancreatic ductal adenocarcinoma”[Title/Abstract] OR PDAC[Title/Abstract]) AND (“metabolic syndrome”[Title/Abstract] OR hyperglyc*[Title/Abstract] OR “fasting glucose”[Title/Abstract] OR insulin*[Title/Abstract] OR “insulin resistance”[Title/Abstract] OR “type 2 diabetes”[Title/Abstract] OR obesity[Title/Abstract] OR “body mass index”[Title/Abstract]) AND (“systematic review”[Publication Type]).

The PROSPERO database was searched using equivalent keyword combinations to identify completed or published systematic reviews addressing metabolic risk factors for pancreatic cancer. The filter was set to “clinical” and “completed”, and the searching code is (pancreatic cancer OR pancreas cancer OR PDAC) AND (metabolic syndrome OR diabetes OR obesity OR insulin resistance OR hyperglycaemia).

The Web of Science search strategy was (“pancreatic cancer” OR “pancreatic neoplasm*” OR “pancreatic ductal adenocarcinoma” OR PDAC) AND (“metabolic syndrome” OR hyperglyc* OR “fasting glucose” OR insulin* OR “insulin resistance” OR “type 2 diabetes” OR obesity OR “body mass index”) AND (“systematic review” OR “meta-analysis” OR “umbrella review”) with certain research area: “Oncology”, “Nutrition Diebetics”, “Endocrinology Metabolism”, “Gastroenterology Hepatology”, “Public Environmental Occupational Health”.

During title and abstract screening, several eligible reviews were identified that evaluated dietary exposures closely related to glucose regulation, insulin resistance, adiposity, and wider metabolic health. These included glycaemic index/load, fructose, meat and protein intake, sweetened beverages, and ultra-processed foods.

### 2.2. Eligibility and Exclusion Criteria

Publications were eligible if they

- Examined incident pancreatic cancer or pancreatic cancer risk in relation to metabolic dysfunction, adiposity-related traits, or dietary exposures considered relevant to metabolic regulation (e.g., obesity, type 2 diabetes, hyperglycaemia, insulin resistance);
- Were systematic reviews, umbrella reviews or meta-analyses reporting quantitative risk estimates where available;
- Were published in English and had an accessible title and abstract;
- Were published between 1 January 2021 and 1 February 2026.

Studies were excluded if they focused solely on treatment efficacy, pharmacological interventions, postoperative outcomes, or prognostic prediction models without etiological relevance, alongside prediction models without an etiological objective, or non-pancreatic cancer outcomes without a pancreatic cancer-specific subgroup. Mechanistic studies were considered for contextual discussion but were not included in the primary evidence synthesis unless accompanied by epidemiological risk assessment. Each review was classified as high-, moderate-, low-, or critically low-quality based on the presence of critical flaws.

For eligibility purposes, related dietary exposures were defined as selected dietary factors with a plausible relationship to glycaemic regulation, insulin response, adiposity, or wider metabolic health. These included meat and protein intake, fructose, sugar-sweetened and artificially sweetened beverages, artificial sweeteners, dietary glycaemic index/load, and ultra-processed foods. The dietary component was intended as a secondary evidence domain rather than a comprehensive review of all nutritional exposures associated with pancreatic cancer.

### 2.3. Study Selection and Data Extraction

All records were imported into reference-management software, Endnote Version 21, and duplicate records were removed before screening. Titles and abstracts were screened against the pre-defined eligibility criteria, followed by full-text assessment of potentially eligible reviews. Reasons for exclusion at the full-text stage were recorded.

For each eligible review, data were extracted using a standardised form. Extracted information included the first author, publication year, review design, databases searched, search period, number and design of included primary studies, study population, exposure definition, pancreatic cancer outcome, effect measure, pooled effect estimate with 95% confidence interval, heterogeneity, publication-bias assessment, risk-of-bias method, and principal conclusions. Pancreatic cancer-specific estimates were extracted when reviews included multiple cancer outcomes. The characteristics of the included reviews are summarised in Table 1, while pancreatic cancer-specific effect estimates for metabolic and adiposity-related exposures and related dietary exposures are presented in Table 2 and Table 3, respectively.

### 2.4. Quality Assessment

The quality of the included systematic reviews was evaluated using A Measurement Tool to Assess Systematic Reviews 2 (AMSTAR-2) [25]. AMSTAR-2 has 16 domains, including critical items such as protocol registration, risk of bias assessment, and publication bias.

The initial AMSTAR-2 assessment was conducted by Junsu Yang and independently verified by co-author Yicong Huang. Disagreements were resolved through discussion and consensus. Reviews were classified as high-quality if there were no or only one non-critical weakness [25]. When reviews had more than one non-critical weakness, they were considered moderate-quality. Low quality indicated that the reviews had one critical flaw with or without non-critical weakness, and critically low quality was defined when the reviews had more than one critical flaw with or without non-critical weakness. The domain-level assessments are presented in Supplement S1.

### 2.5. Evidence Grading

The WCRF CUP framework was applied by considering the totality of evidence for each exposure rather than relying solely on statistical significance [26]. Under this framework, evidence may be classified as convincing, probable, limited/suggestive, limited/no conclusion, or ‘substantial effect on risk unlikely’. Evidence grades were assigned by considering the totality of pancreatic cancer-specific evidence rather than relying solely on statistical significance. The assessment incorporated the quantity and design of the contributing studies, consistency of findings across systematic reviews and meta-analyses, magnitude and precision of pooled effect estimates, between-study heterogeneity, potential publication bias, methodological quality, biological plausibility, and concerns regarding residual confounding or reverse causality.

Where multiple reviews evaluated the same exposure, the evidence was assessed collectively, with greater interpretative weight given to more methodologically robust and recent reviews and to evidence derived predominantly from prospective cohort studies or, where available, Mendelian randomization analyses. Evidence classifications were determined through an overall assessment of these criteria rather than by predefined numerical thresholds. Exposures were classified as probable when broadly consistent positive associations were supported by several prospective studies or evidence syntheses, with reasonably precise estimates and plausible biological mechanisms. Limited/suggestive evidence was assigned when the available findings indicated a possible positive association but were restricted by a small number of studies, modest effect sizes, inconsistency between reviews, methodological limitations, or residual uncertainty. Limited/no conclusion was assigned when the evidence was inconsistent, imprecise, based on insufficient pancreatic cancer-specific data, or otherwise inadequate to support a clear conclusion. No exposure was classified as convincing or as showing a substantial effect on risk unlikely. The WCRF CUP classifications were used to describe the strength and consistency of the available evidence and were not interpreted as establishing causal relationships.

To facilitate visual synthesis, an evidence map was developed using EPPI-Mapper (Version 2.4.5, published 13 August 2025) to summarise the metabolic, adiposity-related, and selected dietary exposures according to their assigned WCRF CUP evidence grades. Rows represent individual exposures, while columns represent the evidence categories observed in this review: limited/no conclusion, limited/suggestive, and probable. Bubble size is proportional to the number of included review–exposure records contributing to each exposure–grade category. The evidence map was intended as a graphical summary of the distribution and relative volume of the available review-level evidence and did not constitute an additional statistical or analytical assessment. Representative pooled effect estimates and the detailed justification for each classification are reported separately in Table 2 and Table 3. The evidence map can be found in Supplement S2.

### 2.6. Assessment of Primary-Study Overlap

Primary-study overlap was assessed at two levels. First, an overall citation matrix was constructed across all 17 included reviews to provide a descriptive measure of cross-review duplication. Second, a domain-restricted analysis was conducted for the two principal evidence domains of adiposity-related exposures and metabolic dysfunction. In each citation matrix, rows represented unique primary studies and columns represented included reviews. A value of 1 indicated that a primary study was included in a review, whereas 0 indicated that it was not included.

The corrected covered area (CCA) was calculated using the total number of primary-study occurrences, the number of unique primary studies, and the number of included reviews [27]. The weighted corrected covered area (wCCA) was additionally calculated to account for differences in the contribution of individual primary studies, with each study weighted according to the square root of its analytical sample size [28]. This weighting reduced the disproportionate influence of very large studies while allowing overlap involving larger studies to contribute more than overlap involving smaller studies. CCA and wCCA values of 0–5%, 6–10%, 11–15%, and >15% were interpreted as indicating slight, moderate, high, and very high overlap, respectively. Dietary-only reviews were excluded from this domain-restricted analysis because they addressed heterogeneous and substantively distinct exposure questions.

## 3. Results

### 3.1. Studies and Characteristics of Included Reviews

**Figure 1:**
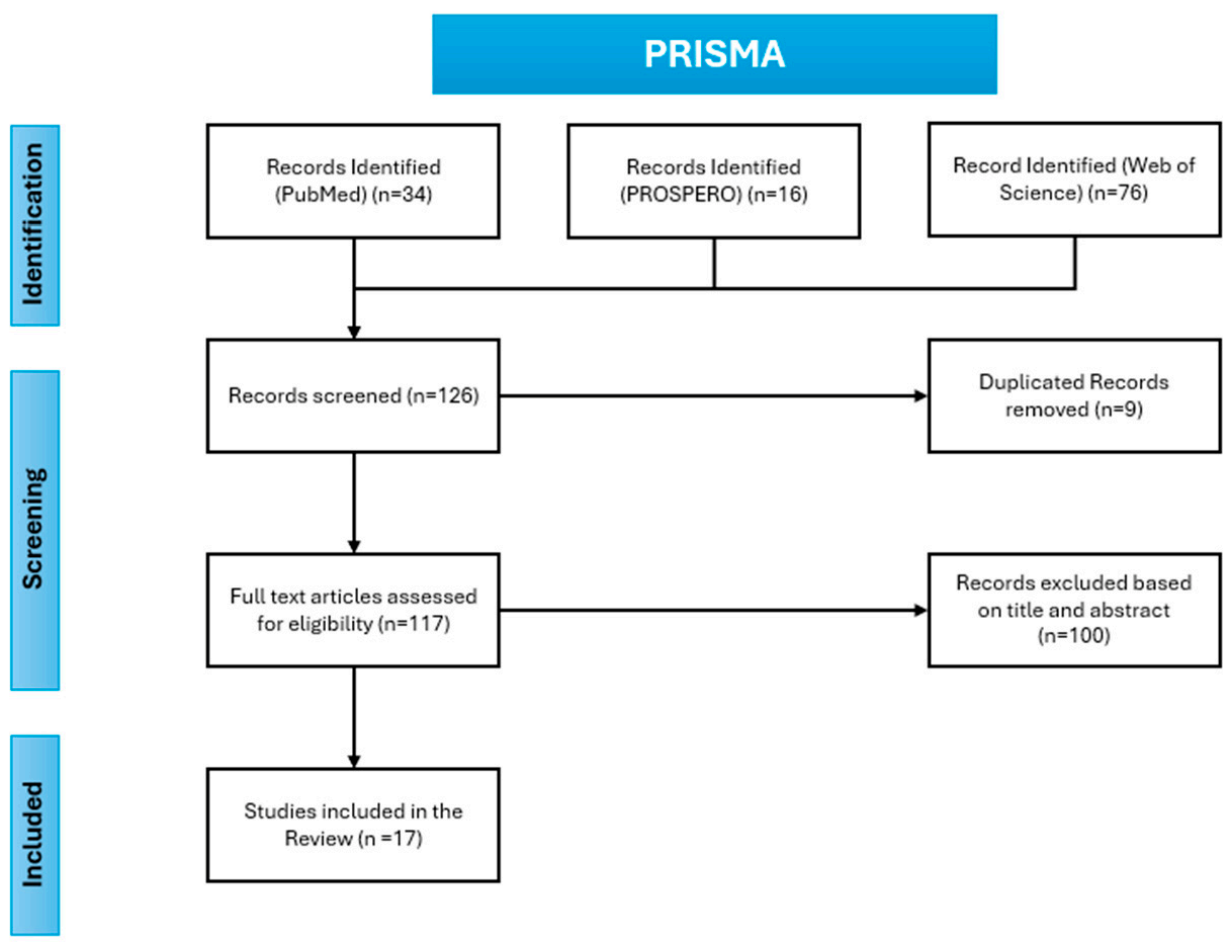
The PRISMA flow diagram of the literature search and selection.

A total of 126 records were identified through database searches, including 34 from PubMed, 16 from the PROSPERO registry and 76 from Web of Science. After removal of duplicates (n = 9) and screening of titles and abstracts, 100 records were excluded for not meeting the eligibility criteria. Seventeen systematic reviews and meta-analyses were ultimately included in the final narrative synthesis (Figure 1).

The included studies comprised eight published systematic reviews or meta-analyses retrieved from PubMed, one completed record identified through PROSPERO and eight systematic reviews from Web of Science. All included publications evaluated metabolic syndrome or related metabolic or dietary exposures in relation to pancreatic cancer risk and are summarised in Table 1. The exposures assessed were heterogeneous but primarily focused on metabolic syndrome as a composite condition, obesity and body mass index (BMI), hyperglycaemia and type 2 diabetes, dyslipidaemia, dietary glycaemic measures, and dietary factors, such as protein intake, meat intake and glycaemic index/load.

Most included reviews synthesised evidence from prospective cohort studies, with several incorporating Mendelian randomization analyses to explore potential causality. Sample sizes of the primary studies underlying these reviews were substantial, frequently exceeding hundreds of thousands of participants, with follow-up durations typically ranging from 5 to over 20 years. Most primary studies were conducted in Europe, North America, and East Asia, reflecting high-income settings with established cancer registries.

The primary outcome across all included reviews was pancreatic cancer incidence. Some studies additionally evaluated mortality outcomes or examined pancreatic cancer as part of broader gastrointestinal cancer analyses. Effect estimates were most commonly reported as risk ratio (RRs), hazard ratios (HRs), or odds ratios (ORs) with corresponding 95% confidence intervals. Heterogeneity across studies was generally moderate, reflecting variation in exposure definitions, adjustment strategies, and population characteristics.

Three reviews were rated as high-quality, with five as moderate, eight reviews as low, and one as critically low. Overall, the included reviews demonstrate substantial diversity in exposure definitions and analytical approaches but consistently focus on metabolic dysfunction as a potential contributor to pancreatic carcinogenesis. A summary of the representative pooled effect estimates and corresponding WCRF/CUP evidence grades for metabolic and dietary exposures is presented in Table 2 and Table 3, respectively.

### 3.2. Methodological Quality of Included Reviews

The methodological quality of the included systematic reviews was assessed using AMSTAR-2. Based on the AMSTAR-2 classification, three reviews were rated as high-quality, five reviews were rated as moderate-quality, eight reviews were rated as low-quality, and one review was rated as critically low-quality [25]. The reviews rated as high-quality include reviews of protein intake, broader dietary factors, and artificially sweetened beverage consumption. The most frequent methodological weaknesses were the absence of a prospectively registered protocol, incomplete reporting of excluded studies, insufficient assessment of publication bias, and limited consideration of primary-study risk of bias when interpreting the findings.

### 3.3. Primary-Study Overlap

Across the 17 included reviews, the citation matrix comprised 148 primary-study occurrences. After accounting for overlap across reviews, these corresponded to 118 unique primary studies. The overall CCA was 1.59%, while the wCCA was 1.40%.

Furthermore, the main-domain citation matrix included seven reviews evaluating adiposity-related exposures or metabolic dysfunction. These reviews contained 58 unique primary studies, while four studies appeared in more than one review. The CCA was 1.15% and the wCCA was 1.81%. These two indicators show a slight overlap at the domain level. However, these figures reflect the overall degree of duplication and should not be interpreted as indicating complete independence among the relevant reviews. The detailed matrix is presented in Supplement S3 (Table S3).

### 3.4. Risk Factors in the Reviews

#### 3.4.1. Adiposity-Related

##### Obesity and Body Mass Index

Obesity and body mass index (BMI) were examined across multiple umbrella reviews and pooled analyses. BMI is a metric related to height and weight and leads to four categories according to the World Health Organization (WHO): underweight (15–19.9), normal weight (20–24.9), overweight (25–29.9) and obese (30–35+) [29]. The umbrella review by Chen et al. included 43 meta-analyses covering 19 cancer types and reported that each 5 kg/m^2^ increase in BMI was associated with an increased risk of pancreatic cancer (RR = 1.10, 95% CI: 1.07–1.14) [10].

Zhong et al., when examining obesity as a component of MetS, did not observe a statistically significant association with pancreatic cancer (RR 1.13, 95% CI 0.96–1.32) [8]. The authors noted that although previous studies have suggested an association between obesity and pancreatic cancer, their pooled estimate did not reach statistical significance [8]. Another study provided a similar result, which indicated RR = 1.04 (95% CI: 1.00–1.09), with four cohort and Mendelian randomization studies [9].

The other review further summarised large prospective cohorts linking elevated BMI with pancreatic cancer risk [11], reinforcing the view that excess adiposity is associated with pancreatic carcinogenesis, though effect sizes vary across populations and analytic models. In this study, both overweight and obesity show a strong relationship with pancreatic cancer, with RRs of 1.237 (95% CI: 1.165–1.314) and 1.306 (95% CI: 1.152–1.480) respectively.

Taken together, obesity appears associated with pancreatic cancer in large, pooled analyses; however, the magnitude of association is modest, and its independence from other metabolic abnormalities remains uncertain.

##### Abdominal Obesity

Compared with overall adiposity, abdominal adiposity, assessed using waist circumference and waist-to-hip ratio, may provide a more accurate indicator of metabolically adverse fat distribution and its potential relationship with pancreatic cancer risk [30]. Abdominal obesity and pancreatic cancer risk were examined using prospective cohort evidence [20]. In the pancreatic cancer subgroup, higher waist circumference was associated with an increased risk of pancreatic cancer based on six prospective studies, with a pooled RR of 1.33 (95% CI: 1.15–1.53). A similar positive association was observed for waist-to-hip ratio, with a pooled RR of 1.42 (95% CI: 1.24–1.63) [20]. These findings suggest that abdominal adiposity indicators were positively associated with pancreatic cancer risk.

#### 3.4.2. Metabolic Dysregulation

##### Metabolic Syndrome

Metabolic syndrome (MetS) is a disease characterised by the co-existence of multiple major risk factors for cardiovascular disease (CVD) and also a risk factor for CVD and type 2 diabetes [31]. The systematic review and meta-analysis by Zhong et al. included both cohort and case–control studies and reported that MetS was significantly associated with an increased risk of pancreatic cancer (RR 1.34, 95% CI 1.23–1.46, *p* < 0.001) [8]. Sex-stratified analyses demonstrated heterogeneity by gender, with a stronger association observed in women (RR 1.64, 95% CI 1.41–1.90) compared with men (RR 1.26, 95% CI 1.03–1.54) [8].

Importantly, component-level analyses indicated that hyperglycaemia (RR 1.55, 95% CI 1.42–1.70), low high-density lipoprotein (HDL) cholesterol (RR 1.24, 95% CI 1.11–1.38), and hypertension (RR 1.10, 95% CI 1.01–1.19) were significantly associated with pancreatic cancer risk, whereas obesity (RR 1.13, 95% CI 0.96–1.32) and hypertriglyceridaemia (RR 0.96, 95% CI 0.87–1.07) were not statistically significant [8].

Zhan et al. conducted a large meta-analysis of 31 prospective cohort studies evaluating MetS and gastrointestinal cancers and suggested a significant association between MetS and pancreatic cancer [9]. Their study further incorporated Mendelian randomization analyses and reported BMI, waist circumference, cholesterol-related traits, and HbA1c as potentially causal factors in pancreatic carcinogenesis [9].

Collectively, these two high-quality syntheses consistently suggest that MetS is associated with a modest but statistically significant increase in pancreatic cancer risk, with hyperglycaemia appearing to be the most influential component.

##### Diabetes Mellitus

Diabetes mellitus was consistently associated with increased pancreatic cancer risk. In the study by Fuentes et al., the pooled estimate was OR 2.30 (95% CI: 2.03–2.62) in case–control studies and HR/RR 2.39 (95% CI: 2.09–2.73) in cohort studies [17]. The association was strongest within the first two years after diabetes diagnosis, with pooled estimates of OR = 3.27 (95% CI: 2.31–4.64) and HR = 4.29 (95% CI: 2.17–8.47) from case–control and cohort studies respectively, and decreased with longer diabetes duration, suggesting that new-onset diabetes may partly reflect reverse causality or early tumour-related metabolic changes [17].

##### Prediabetes

Prediabetes and impaired glucose regulation were evaluated as intermediate metabolic states preceding overt diabetes. In a meta-analysis of five prospective studies comprising eight cohorts, the pooled adjusted effect estimate was 1.40 (95% CI: 1.23–1.59), indicating a higher risk among individuals with prediabetes compared with normoglycaemic individuals [18].

Subgroup analyses suggested that the association may vary by age and geographic region. Studies with participants aged 60 years or older reported a higher effect estimate (ES = 1.83, 95% CI: 1.28–2.62) than studies with younger participants, while Japanese studies showed a higher pooled estimate than studies from the United States [18].

##### Hyperglycaemia

Hyperglycaemia is defined as blood glucose levels above the normal physiological range, typically identified using established clinical thresholds for impaired glucose regulation and diabetes [32]. It emerged as the strongest individual metabolic component associated with pancreatic cancer [8]. Zhong et al. reported a pooled RR of 1.55 (95% CI 1.42–1.70) for hyperglycaemia [8], representing the highest effect estimate among MetS components.

Another article further supported this association using Mendelian randomization analyses involving HbA1c and diabetes-related traits [9]. The convergence of observational meta-analysis and genetic instrumental variable approaches supports the plausibility of a causal interpretation, although causal relationships cannot be established from the evidence synthesised in this review.

Overall, impaired glucose regulation appears to be the most consistently and strongly associated metabolic exposure in relation to pancreatic cancer risk.

##### Dyslipidaemia

Dyslipidaemia is defined as abnormal lipid metabolism characterised by elevated cholesterol or triglycerides, reduced HDL cholesterol, or qualitative alterations in LDL particles [33]. It is a major component of MetS and an important risk factor for CVD [33]. Dyslipidaemia demonstrated heterogeneous associations with pancreatic cancer across lipid components. In a meta-analysis of cohort studies, Zhong et al. reported that reduced high-density lipoprotein (HDL) cholesterol was significantly associated with an increased risk of pancreatic cancer (RR 1.24, 95% CI 1.11–1.38), whereas hypertriglyceridaemia showed no significant association (RR 0.96, 95% CI 0.87–1.07) [8].

Similarly, Zhan et al. evaluated cholesterol-related traits using both prospective cohort studies and Mendelian randomization analyses. Their results indicated that genetically predicted lower HDL cholesterol was associated with pancreatic cancer risk (RR 1.13, 95% CI 1.07–1.18), while triglycerides showed a weaker association with smaller effect estimates (RR 1.07, 95% CI 1.03–1.11) [9].

Overall, the available evidence suggests that lipid abnormalities may be related to pancreatic carcinogenesis, particularly reduced HDL cholesterol. However, the associations are modest and inconsistent across lipid components, and the strength of evidence remains weaker compared with glucose-related metabolic disturbances.

#### 3.4.3. Related Dietary Exposures

##### Dietary Protein

Dietary protein intake was evaluated in the umbrella review by Kühn et al., which identified ten systematic reviews, eight of which included meta-analyses [12]. For pancreatic cancer specifically, higher total protein intake was not associated with risk (RR 0.98, 95% CI 0.63–1.54; I^2^ = 0%) [12,34].

Similarly, animal protein (RR 1.37, 95% CI 0.93–2.01) and plant protein (RR 0.78, 95% CI 0.54–1.14) were not significantly associated [12,34]. The outcome-specific certainty of evidence for pancreatic cancer was rated as very low [12].

Thus, current umbrella-level evidence does not support a significant association between total, animal, or plant protein intake and pancreatic cancer risk.

##### Meat Intake

Meat intake was evaluated in relation to pancreatic cancer risk, with evidence mainly derived from prospective cohort studies. In a pancreatic cancer-specific meta-analysis, red meat intake showed a positive dose–response association, with each 120 g/day increment associated with an increased risk of pancreatic cancer (RR = 1.14, 95% CI: 1.01–1.28) [19]. Processed meat intake was not significantly associated with pancreatic cancer risk, either in the dose–response analysis (RR = 0.99, 95% CI: 0.78–1.25 per 50 g/day) or in the highest versus lowest intake comparison [19]. White meat intake was positively associated with pancreatic cancer risk, with a pooled RR of 1.26 (95% CI: 1.08–1.47) per 100 g/day increment [19].

Consistent with these findings, the umbrella review of dietary factors and pancreatic cancer reported weak evidence for a positive association between red meat intake and pancreatic cancer risk (RR = 1.14, 95% CI: 1.01–1.28), while processed meat intake was not significantly associated with risk (RR = 1.09, 95% CI: 0.96–1.23) [21].

##### Fructose Intake

Fructose intake showed one of strongest dietary signals among the exposures assessed in the umbrella review of prospective evidence. Based on six cohort studies, higher fructose intake was positively associated with pancreatic cancer risk, with a pooled RR of 1.22 (95% CI: 1.09–1.37) [21]. This was the only dietary association in the umbrella review that reached suggestive evidence, while most other dietary exposures were non-significant.

##### Artificially Sweetened Beverages

Artificially sweetened beverages (ASB) and artificial sweeteners were assessed across three included reviews. Evidence from an umbrella review of 11 reports derived from seven systematic reviews and meta-analyses found no significant association between ASB consumption and pancreatic cancer risk. In the pooled cohort analysis, the random-effects estimate was RR = 1.14 (95% CI: 0.93–1.40), with a similar non-significant result under the fixed-effects model (RR = 1.15, 95% CI: 0.97–1.37). Heterogeneity was low to moderate (I^2^ = 25.7%), and there was no clear evidence of small-study effects or excess significance bias [13].

A further dose–response meta-analysis of prospective studies also reported a non-significant pancreatic cancer-specific association for ASB intake, with a pooled RR of 1.10 (95% CI: 0.92–1.31) [24]. Consistent with these findings, broader artificial sweetener intake was not significantly associated with pancreatic cancer when analysed as a non-luminal gastrointestinal cancer outcome (OR = 1.04, 95% CI: 0.95–1.14) [22].

##### Sugar-Sweetened Beverages

Sugar-sweetened beverage (SSB) intake was evaluated in relation to multiple health outcomes, including cancer incidence. In the cancer analysis, evidence was derived from 15 prospective cohort studies, including 1,918,066 participants and 14,166 cancer cases [23]. Across all cancer outcomes, higher SSB intake was associated with a modest increase in overall cancer risk (RR = 1.10, 95% CI: 1.03–1.17), with low between-study heterogeneity [23]. The cancer outcomes included pancreatic cancer, prostate cancer, glioma, endometrial cancer, breast cancer, colorectal cancer, and hepatocellular carcinoma.

For pancreatic cancer specifically, the association was positive in direction but did not reach statistical significance. The pancreatic cancer subgroup estimate was RR = 1.11 (95% CI: 0.95–1.30), indicating that the confidence interval crossed the null value [23]. A dose–response association was observed for total cancer risk, with each 250 mL/day increment in SSB intake associated with increased overall cancer risk (RR = 1.17, 95% CI: 1.04–1.32), but this dose–response estimate was not specific to pancreatic cancer [23].

##### Non-Sugar-Sweetened Beverages

Non-sugar-sweetened beverages (NSSBs) and a range of chronic disease outcomes were assessed in an umbrella review of prospective cohort meta-analyses, synthesising evidence from prospective cohort meta-analyses [14]. For pancreatic cancer, the pooled estimate indicated no statistically significant association with NSSB consumption (HR 1.05, 95% CI 0.89–1.25; I^2^ = 0.00%) [14,24].

##### Dietary Glycaemic Index and Glycaemic Load

Glycaemic index (GI) and glycaemic load (GL) are also two risk factors mentioned in one of the reviews. GI is a unit of measurement of carbohydrate-containing food with a score range from 0 to 100, and GL is the product of GI and the grams of the carbohydrate content in the food to reflect the quality and quantity of dietary carbohydrates [35]. The systematic review evaluating dietary GI and glycaemic load (GL) in relation to pancreatic cancer included several prospective cohort studies and reported different findings [15]. Overall, eight out of ten studies indicated no significant association between dietary GI or GL and pancreatic cancer risk [15]. However, a small number of studies reported significant associations under specific conditions [15]. For example, one cohort study observed a positive association between higher glycaemic load and pancreatic cancer risk during the early follow-up period, while another study reported an inverse association between glycaemic load and pancreatic cancer among women (RR 0.49; *p* for trend = 0.04) [15]. Additionally, an association between insulin load and pancreatic cancer risk was reported in individuals with insulin resistance [15].

Taken together, the available cohort evidence suggests that dietary glycaemic measures do not demonstrate a consistent or robust association with pancreatic cancer risk. Although hyperglycaemia at the metabolic level shows a strong relationship with pancreatic cancer, dietary glycaemic indicators alone appear insufficient to explain this association.

##### Ultra-Processed Food Intake

The systematic review examining ultra-processed food (UPF) consumption evaluated associations between dietary processing levels and cancer risk across multiple observational studies [16]. In the modern food system, UPFs are typically described as industrially manufactured products composed of multiple ingredients, often formulated using refined substances and additives, with poor nutrition, high energy, and high saturated and trans-fat content [36]. Furthermore, high consumption of UPFs could increase the risk of chronic diseases, such as type 2 diabetes, depression and cardiovascular diseases [36]. In the review, nine studies reported significant positive associations between UPF intake and several cancer outcomes after adjustment for potential confounders such as obesity and total energy intake [16]. Specifically, higher UPF consumption was associated with an increased risk of pancreatic cancer, with individuals in the highest intake category showing a significantly elevated risk compared with those in the lowest category (HRQ4 vs. Q1 = 1.49, 95% CI 1.07–2.07) [16].

### 3.5. Strength of Evidence

The strength of evidence was assessed using the WCRF CUP grading criteria [26]. Metabolic syndrome, diabetes mellitus, hyperglycaemia, and abdominal obesity were graded as probable evidence because multiple systematic reviews and meta-analyses reported consistent positive associations, predominantly from prospective cohort studies, supported by moderate to high methodological quality and biological plausibility. Although residual confounding and reverse causality cannot be completely excluded, the consistency of findings across multiple evidence syntheses supported a probable classification.

BMI and general obesity were classified as limited/suggestive because associations were generally positive but modest, with inconsistent findings across reviews and uncertainty regarding independence from other metabolic abnormalities. Prediabetes was also graded as limited/suggestive because the pooled association was positive but based on a small number of studies. Reduced HDL cholesterol was graded as limited/suggestive, whereas hypertriglyceridaemia was classified as limited/no conclusion because available reviews produced inconsistent findings and the evidence was insufficient to support a consistent association with pancreatic cancer risk.

Dietary exposures generally showed weaker evidence. Red meat, white meat, fructose, and ultra-processed food intake were graded as limited/suggestive, while processed meat, protein intake, artificially sweetened beverages, sugar-sweetened beverages, non-sugar-sweetened beverages, and dietary glycaemic index/load were graded as limited/no conclusion because most estimates were non-significant or inconsistent. A summary table of the rationale for each evidence grade can be found in Supplement S4.

**Figure 2:**
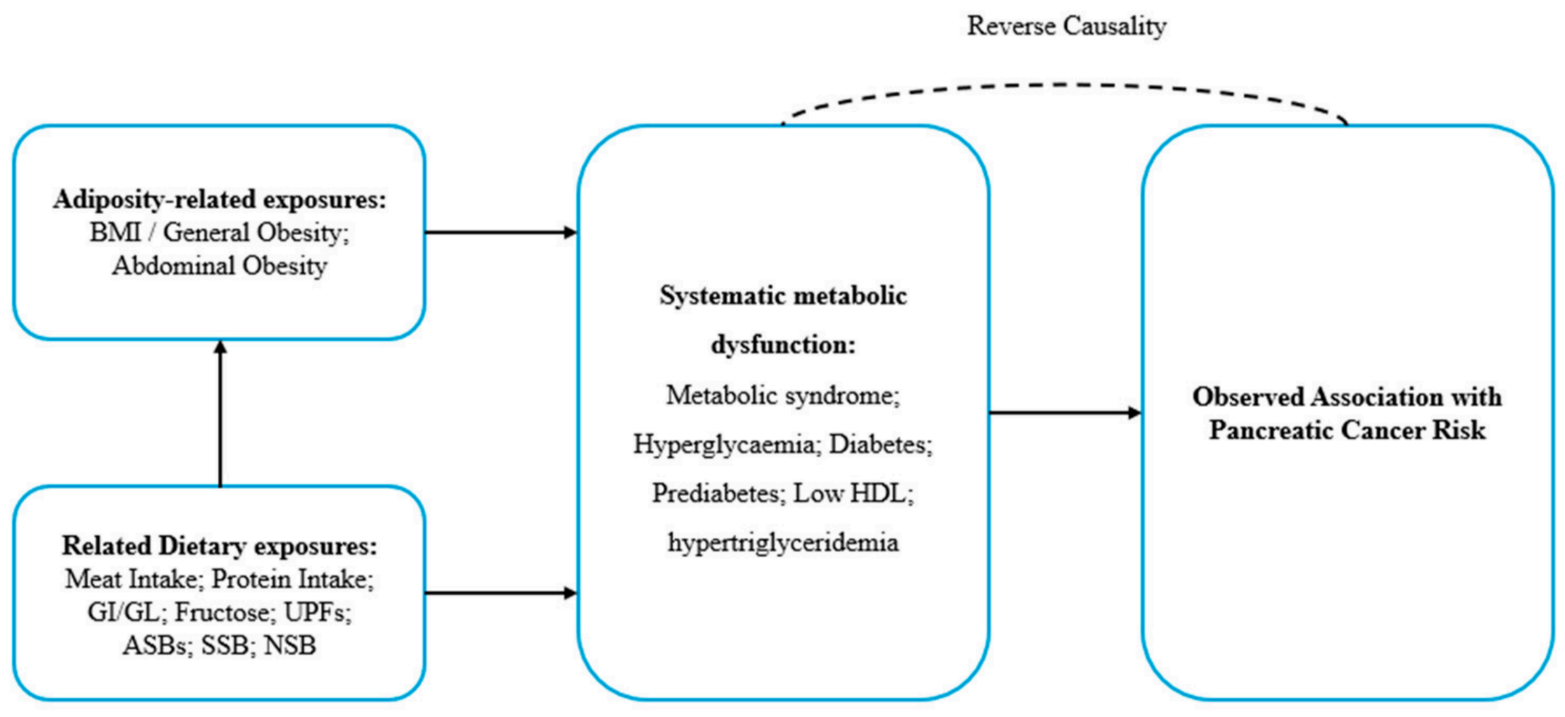
Conceptual framework linking adiposity-related and selected dietary exposures, systemic metabolic dysfunction, and pancreatic cancer risk.

The proposed relationship between dietary and adiposity-related exposures, systemic metabolic dysfunction, pancreatic cancer risk, and reverse causality is summarised in Figure 2.

## 4. Discussion

### 4.1. Main Findings and Interpretation Considering Existing Evidence

This umbrella review synthesised evidence from 17 systematic reviews, umbrella reviews, and meta-analyses evaluating metabolic and related dietary exposures in relation to pancreatic cancer risk. The strongest and most consistent associations were observed for metabolic syndrome, diabetes mellitus, hyperglycaemia, and abdominal adiposity. In contrast, the related dietary exposures examined generally showed weaker, less consistent, or inconclusive associations.

Research in this area used to focus mainly on isolated exposures, such as general obesity, BMI, diabetes status, and individual food groups. However, newer reviews look at abdominal adiposity, metabolic syndrome, prediabetes, age at diabetes onset, and even Mendelian randomization data. The underlying question has shifted: rather than asking whether obesity or diabetes alone matters, researchers are now asking whether glucose dysregulation, fat distribution, lipid abnormalities, and inflammation together mark out a distinct, higher-risk phenotype.

Among the metabolic exposures, impaired glucose regulation appeared to be the most consistent and biologically relevant signal. Hyperglycaemia showed the strongest component-level association with pancreatic cancer, and diabetes mellitus was strongly associated with risk in both cohort and case–control evidence [8,9,17]. Prediabetes also showed a positive association, although the evidence was more limited because it was based on a smaller number of studies [18]. Together, these findings indicate that abnormal glucose metabolism may be an important pathway linking metabolic dysfunction with pancreatic carcinogenesis. However, diabetes-related findings require particularly cautious interpretation because reverse causality remains a major challenge in pancreatic cancer epidemiology. Pancreatic cancer can induce impaired glucose metabolism months or even years before clinical diagnosis [17], meaning that new-onset diabetes may represent an early manifestation of occult malignancy rather than a causal exposure. Consequently, the stronger associations observed shortly after diabetes diagnosis should not be interpreted as evidence of a direct causal relationship. Nevertheless, the persistence of positive associations among individuals with longer-duration diabetes, together with consistent findings for hyperglycaemia, prediabetes, and metabolic syndrome, suggests that chronic metabolic dysfunction may contribute to pancreatic carcinogenesis.

Adiposity-related exposures showed a more complex pattern. General obesity and BMI were associated with pancreatic cancer in some large, pooled analyses, but the magnitude of association was generally modest, and findings were not fully consistent across reviews [8,9,10,11]. This interpretation is also consistent with evidence that abdominal adiposity may be more closely linked to pancreatic cancer risk than overall adiposity, because waist-to-hip ratio captures metabolically adverse fat distribution more effectively and shows a stronger relationship with MetS than BMI [30]. Compared with BMI, abdominal adiposity may better capture metabolically adverse fat accumulation, which is closely related to insulin resistance, chronic inflammation, and dysregulated adipokine signalling [30]. Therefore, excess adiposity may contribute to pancreatic cancer partly through downstream metabolic disturbances rather than through body mass alone.

Evidence for dyslipidaemia was comparatively heterogeneous. Reduced high-density lipoprotein cholesterol showed modest positive associations with pancreatic cancer risk, whereas hypertriglyceridaemia showed inconsistent findings across reviews [8,9]. This suggests that lipid-related abnormalities may contribute to pancreatic carcinogenesis in a more context-dependent manner. Compared with glucose-related exposures, the role of lipid metabolism appears less clearly established and may be influenced by differences in exposure definitions, population characteristics, and adjustment for coexisting metabolic risk factors [9,33].

Dietary exposures generally showed weaker and less consistent evidence. Protein intake, processed meat, ASBs, SSBs, NSSBs, and dietary GI/GL did not show robust or consistent associations with pancreatic cancer risk [12,13,14,15,19,21,22,23,24,34]. Some dietary factors, including red meat, white meat, fructose, and ultra-processed food intake, showed limited/suggestive evidence of positive associations [16,19,21]. However, these findings should be interpreted cautiously because dietary assessments are prone to measurement error, residual confounding, and variation in exposure definitions across studies [14,16,21]. In particular, the evidence for UPF intake was based on limited pancreatic cancer-specific data [16]. Therefore, although some dietary factors may contribute to risk, the current evidence does not support a convincing role for any single dietary exposure.

The contrast between metabolic and dietary findings is important. Dietary exposures may influence pancreatic cancer indirectly through body weight, insulin resistance, glycaemic control, inflammation, and wider metabolic health [9,17,31]. However, the evidence identified in this review was stronger for downstream metabolic states than for individual dietary exposures [8,9,17,18]. This suggests that long-term metabolic dysfunction may be a more measurable and biologically relevant risk profile than isolated dietary components. From a prevention perspective, these findings support a focus on maintaining metabolic health, including healthy body fat distribution, glucose regulation, and diabetes prevention, rather than focusing solely on individual food items.

The stronger and more consistent associations observed for metabolic exposures may reflect both biological plausibility and greater measurement stability. Metabolic abnormalities such as insulin resistance, chronic hyperglycaemia, and systemic inflammation are closely interconnected and have been implicated in pancreatic carcinogenesis through multiple mechanisms, including enhanced cellular proliferation, altered insulin and insulin-like growth factor signalling, oxidative stress, and a pro-inflammatory tumour-promoting microenvironment [37,38,39]. In contrast, individual dietary exposures are often measured with greater error [40,41] and may exert their effects indirectly through long-term influences on body weight, glucose regulation, and metabolic health rather than through direct carcinogenic pathways [42]. Consequently, metabolic markers may better capture the cumulative biological processes most relevant to pancreatic cancer development. At the same time, the interpretation of diabetes-related associations requires caution because of the potential for reverse causality [39]. Pancreatic cancer can induce metabolic disturbances before clinical diagnosis, meaning that diabetes, particularly new-onset diabetes, may represent both a causal risk factor and an early manifestation of occult disease. Nevertheless, the consistency of associations observed across hyperglycaemia, diabetes, prediabetes, and MetS suggests that impaired metabolic regulation is likely to play an important role in pancreatic cancer aetiology beyond reverse-causation effects alone.

The interpretation of these findings should also consider the methodological quality of the included reviews and the evidence grading. AMSTAR-2 assessment indicated that the methodological quality of the included reviews varied, with three reviews rated as high-quality, five as moderate-quality, eight as low-quality, and one as critically low-quality [25]. Several reviews were downgraded because of absent or unclear protocol registration, incomplete reporting of excluded studies, limited assessment of publication bias, or insufficient consideration of risk of bias in interpreting findings. Therefore, findings from low-quality or critically low-quality reviews should be interpreted cautiously. Similarly, no exposure was graded as convincing under the CUP framework, largely because of observational study designs, heterogeneity, possible residual confounding, measurement limitations, and reverse causality for diabetes-related exposures [26].

Overall, this review suggests that pancreatic cancer risk is more strongly associated with integrated metabolic dysfunction than with isolated dietary exposures. The strongest evidence was observed for MetS, diabetes mellitus, hyperglycaemia, and abdominal obesity, while most dietary factors showed limited or inconsistent evidence [8,9,12,13,14,15,16,17,19,20,21,22,23,24,34]. Emerging areas not systematically evaluated in this review include metabolomic profiles such as branched-chain amino acids, more detailed insulin-resistance phenotypes, metabolic inflammation, and environmental metabolic disruptors such as per- and polyfluoroalkyl substances. These factors warrant dedicated future evidence synthesis and prospective investigation. Future research should prioritise large prospective cohorts with repeated metabolic and dietary measurements, better control for confounding, and integration of genetic, metabolomic, and mechanistic evidence to clarify causal pathways and improve risk stratification.

### 4.2. An Integrated Metabolic Dysfunction Framework

Based on the synthesised evidence, we outline an interpretative framework in which dietary exposures and excess adiposity act as upstream influences, with insulin resistance, chronic hyperglycaemia, diabetes, dyslipidaemia, and low-grade inflammation constituting more proximal features of systemic metabolic dysfunction. These processes rarely operate in isolation, which may help explain why composite metabolic phenotypes were more consistently associated with pancreatic cancer risk than individual dietary exposures.

The framework is offered as a means of organising existing evidence, not as a validated causal model. Much of the underlying evidence is observational, and the proposed pathways cannot be dissociated from residual confounding, measurement error, and reverse causality.

### 4.3. Limitations

This review has several limitations. First, although 17 reviews were included, the number of pancreatic cancer-specific primary studies was limited for several exposures. Some associations were based on only a small number of cohort studies or on subgroup estimates within broader cancer, gastrointestinal cancer, or chronic disease reviews. This limits the statistical power and robustness of the evidence, particularly for prediabetes, lipid-related exposures, ultra-processed food intake, and some beverage-related exposures. The evidence on ultra-processed food intake came from a single pancreatic cancer-specific cohort, and beverage-related exposures were synthesised repeatedly across reviews that likely drew on overlapping primary cohorts. Associations for red meat, white meat, and fructose, though statistically significant, were modest in magnitude and vulnerable to dietary measurement error, residual confounding, multiple comparisons, and selective reporting.

Second, there was substantial heterogeneity across the included reviews. Studies differed in exposure definitions, outcome definitions, population characteristics, follow-up duration, and adjustment strategies. For example, adiposity was assessed using BMI, overweight/obesity categories, waist circumference, or waist-to-hip ratio, while dietary exposures varied widely in measurement methods and intake categories. These differences may partly explain the inconsistent findings observed across some exposures.

Third, most evidence was derived from observational studies. Although many primary studies were prospective cohorts, residual confounding cannot be excluded. This is particularly important for dietary exposures, where measurement error, changes in diet over time, and confounding by smoking, alcohol intake, BMI, diabetes, and socioeconomic factors may influence the observed associations. Therefore, null or weak findings for dietary factors should not be interpreted as definitive evidence of no effect.

Fourth, reverse causality remains a major concern for diabetes, hyperglycaemia, and related metabolic traits. Pancreatic cancer can induce metabolic changes before clinical diagnosis, and new-onset diabetes may represent an early manifestation of occult pancreatic cancer rather than a causal exposure. This limits causal interpretation, particularly in studies without sufficient temporal separation between diabetes onset and pancreatic cancer diagnosis.

Fifth, primary-study overlap may have affected the apparent independence of the evidence. Overlap was formally assessed within the two principal evidence domains of adiposity-related exposures and metabolic dysfunction. The citation matrix included seven reviews, comprising 58 unique primary studies and 62 primary-study occurrences, with four studies appearing in more than one review. The corrected covered area (CCA) was 1.15%, indicating limited overall overlap based on the frequency of duplicated primary studies, while the weighted corrected covered area (wCCA) was slightly higher at 1.81%, indicating that accounting for the relative contribution of overlapping evidence did not materially alter the assessment. Taken together, these findings suggest that extensive duplication of the underlying evidence was unlikely to explain the overall consistency observed across these reviews. Nevertheless, the overlap analysis was conducted at the combined domain level and may therefore obscure more concentrated duplication within individual exposure categories, such as BMI, obesity, or components of metabolic syndrome. Dietary-only reviews were excluded from this analysis because they addressed heterogeneous and substantively distinct exposure questions. Accordingly, the low CCA and wCCA values indicate limited overall duplication within the principal evidence domains, but they do not demonstrate complete independence among the contributing reviews. Concordance between reviews should therefore not be interpreted as fully independent replication.

Sixth, the evidence regarding early-onset pancreatic ductal adenocarcinoma (PDAC) remains particularly limited. The systematic review examining early-onset PDAC risk factors relied primarily on a single case and control study design [43]. Moreover, exposures such as body mass index were stratified into multiple subgroups across different life stages, resulting in relatively small sample sizes within each analytic category [43]. This extensive stratification substantially reduced statistical power and increased the uncertainty of the reported estimates. As a result, the findings from early-onset PDAC analyses should be interpreted with caution and may currently serve only as preliminary reference evidence rather than definitive conclusions.

Finally, the CUP grading applied in this review required judgement because several exposures were supported by mixed evidence from different study designs or from subgroup analyses. Although the grading provides a structured framework for evaluating evidence strength, the classifications should be interpreted as evidence-based judgements rather than definitive causal conclusions [25,26].

These limitations highlight the need for larger prospective cohort studies and well-powered meta-analyses to clarify the role of metabolic and dietary exposures in pancreatic cancer development. Finally, every effort was made to verify effect estimates, study characteristics, and reference citations against the original publications included within the eligible reviews. However, as this umbrella review relied on previously published evidence syntheses, any reporting inaccuracies present in the source reviews may have been carried forward despite careful checking.

## 5. Conclusions

In summary, the evidence synthesised in this umbrella review suggests that pancreatic cancer risk is more strongly associated with systemic metabolic dysfunction than with individual related dietary exposures. Although diabetes mellitus demonstrated one of the strongest associations with pancreatic cancer risk, this finding should be interpreted cautiously because of the potential for reverse causality, particularly in individuals with new-onset diabetes, which may represent an early manifestation of undiagnosed pancreatic cancer. Hyperglycaemia, metabolic syndrome, and abdominal obesity also demonstrated the most consistent associations and were graded as probable evidence, whereas general obesity, prediabetes, and reduced HDL cholesterol were supported by limited/suggestive evidence. Most dietary exposures showed limited, inconsistent, or inconclusive associations with pancreatic cancer risk, although red meat, white meat, fructose, and ultra-processed food intake were classified as limited/suggestive. Notably, no exposure met the criteria for convincing evidence. Collectively, these findings suggest that maintaining metabolic health, including optimal glucose regulation and the prevention of abdominal adiposity, may help identify individuals at increased risk of pancreatic cancer and could contribute to risk reduction, although causal relationships require further investigation. Future research should prioritise large prospective studies incorporating repeated exposure assessments, biomarker measurements, genetic and causal inference branched-chain amino-acid approaches, and mechanistic investigations to further clarify causal pathways and strengthen the evidence base.

## Figures and Tables

**Table 1:** Summary table of the reviews included in this review.

Author/Year	Number of Studies	Exposures	Outcomes	Quality
Zhong 2023 [8]	7 cohort + 2 case–control studies	Metabolic syndrome + components	Pancreatic cancer	Low
Zhan 2024 [9]	31 prospective cohorts + Mendelian randomization studies	MetS, BMI, HbA1c, lipids	Gastrointestinal cancers including pancreatic cancer	Moderate
Chen 2023 [10]	43 meta-analyses	BMI	Multiple cancers including pancreatic	Critical Low
Ren 2025 [11]	39 prospective cohort studies	Overweight/obesity	Digestive cancers incl. pancreatic	Moderate
Kühn 2024 [12]	10 reviews (8 meta-analyses)	Total/animal/plant protein	Pancreatic cancer	High
Diaz 2023 [13]	11 systematic reviews and meta-analyses	Artificially sweetened beverages	Multiple cancers including pancreatic cancer	Moderate
Beigrezaei 2024 [14]	6 systematic reviews and meta-analyses	Non-sugar-sweetened beverages	Chronic diseases including pancreatic cancer	Moderate
Marbini 2021 [15]	53 cohort studies	Glycaemic index/load	Pancreatic cancer	Low
Isaksen 2023 [16]	21 studies	Ultra-processed food intake	Cancer outcomes including pancreatic cancer	Low
Fuentes A. 2025 [17]	23 studies	Diabetes mellitus	Pancreatic cancer	Moderate
Jain 2025 [18]	5 studies	Prediabetes	Pancreatic cancer	Low
Kim, Y. 2023 [19]	20 prospective cohort studies	Red/processed/white meat	Pancreatic cancer	Low
Li et al. 2023 [20]	43 prospective cohort studies	Abdominal obesity: waist circumference/waist-to-hip ratio	Digestive system cancers including pancreatic cancer	Low
Qin et al. 2023 [21]	41 meta-analyses	Dietary factors	Pancreatic cancer	High
Tepler et al. 2021 [22]	8 studies	Artificial sweeteners	Gastrointestinal cancers including pancreatic cancer	Low
Wang Y. 2022 [23]	32 prospective studies	Sugar-sweetened beverages	Multiple diseases including pancreatic cancer	Low
Yin et al. 2022 [24]	14 articles	Artificially sweetened beverages	Multiple cancers including pancreatic cancer	High

**Table 2:** Detailed table of the reviews and the strength of evidence by metabolic risk factors included in this review.

Domain	Risk Factor	Author and Year	Number of Studies (Related to Pancreatic Cancer by Risk Factor)	Result	Strength of Evidence
Adiposity-related	BMI and Obesity	Zhong 2023 [8]	5 cohort studies	Obesity: RR: 1.13 (95% CI: 0.96–1.32, *p* = 0.151)	Limited/suggestive
		Zhan 2024 [9]	4 cohorts and Mendelian randomization studies	Obesity: RR: 1.04 (95% CI: 1.00–1.09, *p* = 0.073)	
		Chen 2023 [10]	1 meta-analysis with 23 cohort studies	Every 5 kg/m^2^ Increase in BMI: RR: 1.10 (95% CI: 1.07–1.14)	
		Ren 2025 [11]	4 studies explored the association between overweight/obesity and DSCs in men/6 studies addressed this relationship in women	Overweight: RR: 1.237 (95% CI: 1.165–1.314); Obesity: RR: 1.306 (95% CI: 1.152–1.480)	
	Abdominal obesity	Li 2023 [20]	6 prospective studies	Waist circumference: RR: 1.33 (95% CI: 1.15–1.53); Waist-to-hip ratio: RR: 1.42 (95% CI: 1.24–1.63)	Probable
Metabolic dysregulation	Metabolic syndrome	Zhong 2023 [8]	7 cohort + 2 case–control studies	RR: 1.34 (95% CI: 1.23–1.46, *p* < 0.001); By gender: men: RR 1.26, (95% CI 1.03–1.54, *p* = 0.022); women: RR 1.64, (95% CI 1.41–1.90, *p* < 0.001)	Probable
		Zhan 2024 [9]	6 cohorts and Mendelian randomization studies	RR: 1.25 (95% CI: 1.20–1.30)	Probable
	Diabetes mellitus	Fuentes 2025 [17]	9 cohort and 14 case–control studies	Cohort: HR: 2.39 (95% CI: 2.09–2.73); Case–control: OR: 2.3 (95% CI: 2.03–2.62)	Probable
	Prediabetes	Jain 2025 [18]	5 studies	Adjusted ES: 1.40 (95% CI: 1.23–1.59, *p* < 0.01)	Limited/suggestive
	Hyperglycaemia	Zhong 2023 [8]	4 cohort studies	RR: 1.55 (95% CI: 1.42–1.70, *p* < 0.001)	Probable
		Zhan 2024 [9]	4 cohorts and Mendelian randomization studies	RR: 1.30 (95% CI: 1.14–1.47, *p* < 0.001)	
	Low high-density lipoprotein cholesterol	Zhan 2024 [9]	3 cohorts and Mendelian randomization studies	Reduced HDL: RR: 1.13 (95% CI: 1.07–1.18, *p* < 0.001)	Limited/suggestive
		Zhong 2023 [8]	4 cohort studies	low high-density lipoprotein cholesterol: RR: 1.24 (95% CI: 1.11–1.38, *p* < 0.001)	
	Hypertriglyceridemia	Zhong 2023 [8]	4 cohort studies	RR: 0.96 (95% CI: 0.87–1.07, *p* = 0.486)	Limited/no conclusion
		Zhan 2024 [9]	4 cohorts and Mendelian randomization studies	RR: 1.07 (95% CI: 1.03–1.11, *p* < 0.001)	

**Table 3:** Detailed table of the reviews and the strength of evidence by dietary risk factors included in this review.

Domain	Risk Factor	Author and Year	Number of Studies (Related to Pancreatic Cancer by Risk Factor)	Result	Strength of Evidence
Dietary factor	Protein intake	Kühn 2024 [12]	1 systematic review	Total protein: RR: 0.98 (95% CI: 0.63–1.54); Animal protein: RR: 1.37 (95% CI: 0.93–2.01); Plant protein: RR 0.78 (95% CI: 0.54–1.14)	Limited/no conclusion
	Meat intake	Kim 2023 [19]	20 prospective cohort studies	Red meat (120 g/day): RR: 1.14 (95% CI: 1.01–1.28); Processed meat (50 g/day): RR: 0.99 (95% CI: 0.78–1.25); White meat (100 g/day): RR: 1.26 (95% CI: 1.08–1.47);	Limited/suggestive Limited/no conclusion Limited/suggestive
		Qin 2023 [21]	1 study with 29 cohort studies	Red meat: RR: 1.14 (95% CI: 1.01–1.28); Processed meat: RR: 1.09 (95% CI: 0.96–1.23)	Limited/suggestive Limited/no conclusion
	Fructose	Qin 2023 [21]	1 study with 6 cohort studies	RR: 1.22 (95% CI: 1.09–1.37)	Limited/suggestive
	Artificially sweetened beverages/sweeteners	Diaz 2023 [13]	5 meta-analyses (cohort and case–control studies)	Random effects: RR: 1.14 (95% CI: 0.93–1.40, *p* = 0.215); Fixed-effect: RR: 1.15 (95% CI: 0.97–1.37, *p* = 0.102)	Limited/no conclusion
		Tepler 2021 [22]	6 studies	OR: 1.04 (95% CI: 0.95 –1.14)	
		Yin 2022 [24]	4 studies	RR: 1.10 (95% CI: 0.92–1.31)	
	Sugar-sweetened beverages	Wang 2022 [23]	5 studies	RR: 1.11 (95% CI: 0.95–1.30)	Limited/no conclusion
	Non-sugar-sweetened beverages	Beigrezaei 2024 [14]	1 Review	HR: 1.05 (95% CI: 0.89–1.25; I^2^: 0.00%)	Limited/no conclusion
	Glycaemic index/load (GI/GL)	Marbini 2021 [15]	10 studies	Two of them show a strong association between glycaemic index/load (GI/GL) and pancreatic cancer, whereas the rest indicate that there is no association between GI/GL and pancreatic cancer	Limited/no conclusion
	Ultra-processed foods	Isaksen 2023 [16]	21 studies	HR (Q4 vs. Q1) = 1.49, 95% CI 1.07–2.07	Limited/suggestive

## Data Availability

No new data were created or analyzed in this study. Data sharing is not applicable to this article.

## Supplementary Materials

The following supporting information can be downloaded at https://www.mdpi.com/article/10.3390/ijms27177707/s1.

## Abbreviations

The following abbreviations are used in this manuscript:

BMI	body mass index
MetS	Metabolic syndrome
CVD	cardiovascular disease
RR	risk ratio
HR	hazard ratio
OR	odds ratio
HDL	high-density lipoprotein
ASB	artificially sweetened beverages
SSB	sugar-sweetened beverage
NSSB	non-sugar-sweetened beverages
GI	glycaemic index
GL	glycaemic load
UPF	ultra-processed food
PDAC	pancreatic ductal adenocarcinoma
CI	confident interval
CUP	Continuous Update Project
WCRF	World Cancer Research Fund
CCA	Corrected Covered Area
AMSTAR-2	Assess Systematic Reviews 2
PRISMA	Preferred Reporting Items for Systematic Reviews and Meta-Analyses
