# Supplementary material for: Metabolic Dysfunction and Related Dietary Exposures in Relation to Pancreatic Cancer Risk: A Review of the Evidence
Source: Int J Mol Sci. 2026 Aug 28;27(17):7707. doi: 10.3390/ijms27177707 (PMC13566534; doi:10.3390/ijms27177707)
Supplement: Supplementary file 1 [file ijms-27-07707-s001.zip › ijms-4396158-supplementaryS1.pdf]

Supplement S1. Domain-level AMSTAR-2 assessment of included reviews.

ID	REVIEW	EXPOSURE/DOMAIN	Q1	Q2*	Q3	Q4*	Q5	Q6	Q7*	Q8	Q9*	Q10	Q11*	Q12	Q13*	Q14	Q15*	Q16	CRITICAL FLAWS	NON-CRITICAL WEAKNESSES	CALCULATED RATING
1	Zhong 2023 [8]	Metabolic syndrome and components	Y	Y	N	PY	Y	Y	N	Y	PY	N	Y	N	Y	Y	Y	Y	1	5	Low
2	Zhan 2024 [9]	Metabolic syndrome/ components and Mendelian randomization	Y	Y	Y	PY	Y	Y	PY	Y	PY	N	Y	N	Y	Y	Y	Y	0	5	Moderate
3	Chen 2023 [27]	BMI/general obesity	Y	N	N	PY	N	Y	N	Y	Y	N	Y	N	Y	Y	Y	Y	2	5	Critically low
4	Ren 2025 [11]	Overweight/obesity	Y	Y	Y	PY	N	Y	Y	Y	PY	N	Y	N	Y	Y	Y	Y	0	5	Moderate
5	Kühn 2024 [12]	Protein intake	Y	Y	Y	Y	Y	Y	Y	Y	Y	N	NMA	NMA	Y	Y	NMA	Y	0	1	High
6	Diaz 2023 [13]	Artificially sweetened beverages	Y	PY	Y	PY	Y	Y	PY	Y	Y	N	Y	Y	Y	Y	Y	Y	0	4	Moderate
7	Beigrezaei 2025 [14]	Non-sugar-sweetened beverages	Y	Y	Y	PY	Y	Y	PY	Y	Y	N	Y	Y	Y	Y	Y	Y	0	3	Moderate
8	Marbini 2021 [15]	Glycaemic index/load	Y	PY	Y	PY	Y	Y	PY	Y	N	N	NMA	NMA	Y	Y	NMA	Y	1	4	Low
9	Isaksen 2023 [16]	Ultra-processed food intake	Y	N	Y	PY	Y	Y	PY	Y	PY	N	Y	N	Y	Y	Y	Y	1	5	Low
10	Fuentes 2025 [30]	Diabetes mellitus	Y	Y	Y	PY	N	Y	PY	Y	PY	N	Y	N	Y	Y	Y	Y	0	6	Moderate
11	Jain 2025 [31]	Prediabetes	Y	N	Y	PY	Y	N	PY	Y	PY	N	Y	N	Y	Y	PY	Y	1	7	Low
12	Kim 2023[17]	Red, processed and white meat	Y	N	Y	PY	N	N	PY	Y	PY	N	Y	N	Y	Y	Y	Y	1	7	Low
13	Li 2023 [18]	Abdominal obesity	Y	N	Y	PY	Y	Y	PY	Y	PY	N	Y	N	Y	Y	Y	Y	1	5	Low
14	Qin 2023 [19]	Dietary factors	Y	Y	Y	Y	Y	Y	Y	Y	Y	N	Y	Y	Y	Y	Y	Y	0	1	High
15	Tepler 2021 [20]	Artificial sweeteners	Y	N	Y	Y	Y	Y	PY	Y	PY	N	Y	N	Y	Y	Y	Y	1	4	Low
16	Wang 2022 [21]	Sugar-sweetened beverages	Y	PY	Y	Y	Y	Y	N	Y	PY	N	Y	N	Y	Y	Y	Y	1	4	Low
17	Yin 2022 [22]	Artificially sweetened beverages	Y	Y	Y	Y	Y	Y	Y	Y	Y	N	Y	Y	Y	Y	Y	Y	0	1	High

Abbreviations: Y, Yes; PY, Partial Yes; N, No; NMA, no meta-analysis conducted. Asterisks indicate AMSTAR-2 critical domains.
