# Supplementary material for: Metabolic Dysfunction and Related Dietary Exposures in Relation to Pancreatic Cancer Risk: A Review of the Evidence
Source: Int J Mol Sci. 2026 Aug 28;27(17):7707. doi: 10.3390/ijms27177707 (PMC13566534; doi:10.3390/ijms27177707)

Supplement S2. Evidence map of metabolic dysfunction and related dietary exposures

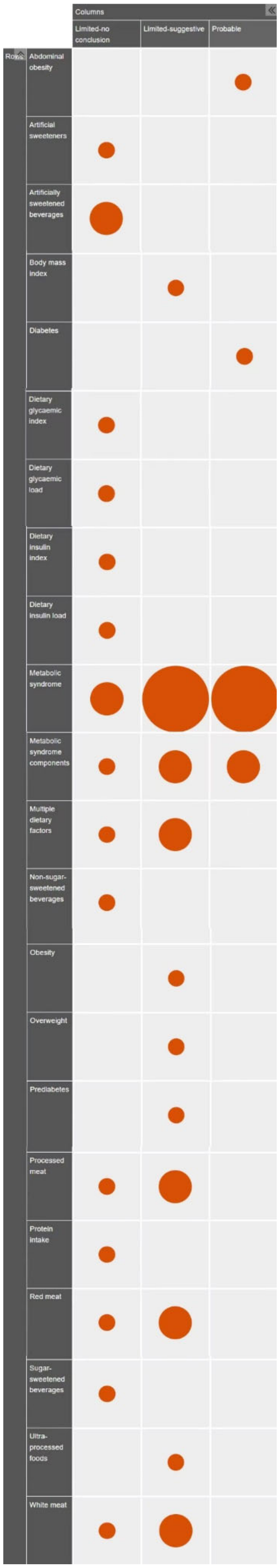

Supplement: Supplementary file 1 [file ijms-27-07707-s001.zip › ijms-4396158-supplementaryS2.pdf]
