# Supplementary material for: Metabolic Dysfunction and Related Dietary Exposures in Relation to Pancreatic Cancer Risk: A Review of the Evidence
Source: Int J Mol Sci. 2026 Aug 28;27(17):7707. doi: 10.3390/ijms27177707 (PMC13566534; doi:10.3390/ijms27177707)
Supplement: Supplementary file 1 [file ijms-27-07707-s001.zip › ijms-4396158-supplementaryS3.pdf]

Supplement S3. Primary-study Citation Matrix for CCA/wCCA Assessment

A value of 1 indicates that a primary study was included in the corresponding review. Only studies contributing to at least one review shown in each panel are displayed. Blank cells indicate non-inclusion. Sample size is the value used for the wCCA weighting step.

Table S3a, Panel A. Reviews: Zhong 2023, Zhan 2024, Chen 2023, Ren 2025, Kühn 2024, and Diaz 2023.

Study	*Zhong 2023*	*Zhan 2024*	*Chen 2023*	*Ren 2025*	*Kühn 2024*	*Diaz 2023*	*Sample Size*
Antonio Russo; 2008	1						16,677
Manami Inoue; 2009	1						27,724
Valentina Rosato; 2011	1						978
Bin Xia; 2020	1	1					475,078
Sung Keun Park; 2020	1						222,838
HyeSoo Chung; 2021	1						347,434
Joo-Hyun Park; 2022	1	1					8,203,492
Joseph A; 2022	1						366,494
Tomàs LópezJiménez; 2022	1						183,284
Johansen D; 2010		1		1			33,346
Aune 2012			1				3,236,625
Shyam; 2021				1			35,364
Oh; 2005				1			781,283
Liu; 2016				1			68,253
da Silva; 2018				1			135,708
Jee; 2008				1			1,213,829
Samanic; 2006				1			362,552
Hoyt; 2022				1			145,489
Lukanova; 2006				1			68,786
Stolzenberg; 2008				1			495,035
Stolzenberg; 2013				1			501,698

Andreotti; 2010	1			67,947
Jiao; 2010	1			943,759
Rapp; 2005	1			145,931
Larsson; 2005	1			83,053
Jung; 2019	1			10,962,385
Alzahrani; 2022		1		2,015,224
Zhang; 2022		1		223,424
Llaha; 2021			1	1,991,179

Table S3a, Panel B. Reviews: Beigrezaei 2025, Marbini 2021, Isaksen 2023, Fuentes 2025, Jain 2025, and Kim 2023.

Study	*Beigrezaei 2025*	*Marbini 2021*	*Isaksen 2023*	*Fuentes 2025*	*Jain 2025*	*Kim 2023*	*Sample Size*
Manami Inoue; 2009						1	27,724
Jiao; 2010		1					943,759
Larsson; 2005						1	83,053
Zhang; 2022	1						223,424
Yin; 2022	1						699,434
Simon; 2010		1					161,809
Meinhold; 2010		1					109,175
George; 2009		1					446,177
Heinen; 2008		1				1	120,852
Northlings; 2007		1				1	162,150
Patel; 2007		1					124,807
Silvera; 2005		1					49,613
Johnson; 2005		1					34,699
Michaud; 2002		1				1	88,802
Bao; 2011		1					132,887
Zhong; 2022			1				98,265
Lu; 2015				1			5,529
Walker; 2015				1			1,409
Antwl; 2016				1			2,573
Ben; 2016				1			756
Olson; 2016				1			973
Zheng; 2016				1			504
Wang; 2016				1			1,163

Valente; 2017	1			804
Li; 2018	1			687
Petrusel; 2020	1			591
Farias; 2020	1			2,161
Dayem; 2021	1			5,320
Van Trant; 2021	1			384
Tan; 2023	1			289,356
Yuan; 2020	1			159,025
Park BK; 2022	1			7,445,944
Lee DY, 2018	1			4,945,886
Huang BZ, 2020	1		1	1,499,627
Er KC, 2016	1			699,115
White MJ, 2023	1			2,561,684
Setiawan VW, 2018	1			48,995
Safadi H, 2024	1			3,681,774
de Jong RGPJ, 2017	1			34,038
Jacobson; 2021		1		899
Rentsch; 2020		1		378,253
Koo; 2019		1		5,392,684
Keum; 2017		1		319
Goto; 2016		1		16,347
Zheng; 1993			1	17,633
Coughlin; 2000			1	1,102,308
Isaksson; 2002			1	21,884
Stolzenberg-Solomon; 2002			1	27,111
Khan; 2004			1	3,158

Lin; 2006	1	110,792
Rohrman; 2013	1	477,202
Ghorbani; 2016	1	50,045
Taunk; 2016	1	322,846
McCullough; 2018	1	138,266
Pang; 2018	1	510,314
Knuppel; 2020	1	474,996
Petrick; 2020	1	52,706

Table S3a, Panel C. Reviews: Li 2023, Qin 2023, Tepler 2021, Wang 2022, and Yin 2022.

Study	Li 2023	Qin 2023	Tepler 2021	Wang 2022	Yin 2022	Sample Size
Manami Inoue; 2009	1					27,724
Aune 2012		1				3,236,625
Liu; 2016		1				68,253
Larsson; 2005				1		83,053
Zhang; 2022		1				223,424
Llaha; 2021		1				1,991,179
Bao; 2011	1		1	1	1	132,887
Zheng; 2016		1				504
Wang; 2016		1				1,163
McCullough; 2018	1					138,266
Navarrete-Muñoz; 2016	1		1		1	477,199
Schernhammer; 2005	1		1	1	1	138,158
Zhao; 2018		1				3,338,996
Darooghegi; 2021		1				928,461
Bae; 2009		1				1,478,929
Paluszkiewicz; 2012		1				539,706
Jiang; 2019		1				1,776,968
Zhao; 2017		1				6,006,840
Gao; 2022		1				1,258,913
Arafa; 2021		1				2,809,013
Chen; 2014		1				853,419
Filippini; 2020		1				319,741
Gallus; 2011		1	1			929,709
Nie; 2016		1				1,869,416

Zhou; 2019	1			856,794
Shen; 2015	1			1,064,123
Michels; 2021	1			683,363
Wang; 2020	1			148,640
Kim; 2020	1			762,238
Yao; 2015	1			2,878,123
Nucci; 2021	1			332,690
Ko; 2014	1			525,095
Li; 2017	1			101,104
Huang; 2016	1			216,272
Wei; 2020	1			615,904
Hua; 2016	1			278,000
Fu; 2021	1			1,002,034
Chen; 2016	1			198,298
Filippini; 2020	1			598,934
Lei; 2016	1			598,866
Picetti; 2022	1			32,251
Guo; 2021	1			634,705
Yu; 2012	1			141,903
Cai; 2019	1			2,433,416
Norell; 1986		1		234
Chan; 2009		1		2,227
Mueller; 2010			1	60,524

Table S3b. Metabolic-domain Citation Matrix

This table reproduces the metabolic-domain subset in Sheet2 of the supplied workbook. Here, 1 indicates inclusion and 0 indicates non-inclusion.

Study	*Zhong 2023*	*Zhan 2024*	*Chen 2023*	*Ren 2025*	*Fuentes 2025*	*Jain 2025*	*Li 2023*	*Sample Size*
Antonio Russo; 2008	1	0	0	0	0	0	0	16,677
Manami Inoue; 2009	1	0	0	0	0	0	1	27,724
Valentina Rosato; 2011	1	0	0	0	0	0	0	978
Bin Xia; 2020	1	1	0	0	0	0	0	475,078
Sung Keun Park; 2020	1	0	0	0	0	0	0	222,838
HyeSoo Chung; 2021	1	0	0	0	0	0	0	347,434
Joo-Hyun Park; 2022	1	1	0	0	0	0	0	8,203,492
Joseph A; 2022	1	0	0	0	0	0	0	366,494
Tomàs LópezJiménez; 2022	1	0	0	0	0	0	0	183,284
Johansen D; 2010	0	1	0	1	0	0	0	33,346
Aune 2012	0	0	1	0	0	0	0	3,236,625
Shyam; 2021	0	0	0	1	0	0	0	35,364
Oh; 2005	0	0	0	1	0	0	0	781,283
Liu; 2016	0	0	0	1	0	0	0	68,253
da Silva; 2018	0	0	0	1	0	0	0	135,708
Jee; 2008	0	0	0	1	0	0	0	1,213,829
Samanic; 2006	0	0	0	1	0	0	0	362,552
Hoyt; 2022	0	0	0	1	0	0	0	145,489
Lukanova; 2006	0	0	0	1	0	0	0	68,786
Stolzenberg; 2008	0	0	0	1	0	0	0	495,035
Stolzenberg; 2013	0	0	0	1	0	0	0	501,698
Andreotti; 2010	0	0	0	1	0	0	0	67,947
Jiao; 2010	0	0	0	1	0	0	0	943,759
Rapp; 2005	0	0	0	1	0	0	0	145,931
Larsson; 2005	0	0	0	1	0	0	0	83,053

Jung; 2019	0	0	0	1	0	0	0	10,962,385
Bao; 2011	0	0	0	0	0	0	1	132,887
Lu; 2015	0	0	0	0	1	0	0	5,529
Walker; 2015	0	0	0	0	1	0	0	1,409
Antwl; 2016	0	0	0	0	1	0	0	2,573
Ben; 2016	0	0	0	0	1	0	0	756
Olson; 2016	0	0	0	0	1	0	0	973
Zheng; 2016	0	0	0	0	1	0	0	504
Wang; 2016	0	0	0	0	1	0	0	1,163
Valente; 2017	0	0	0	0	1	0	0	804
Li; 2018	0	0	0	0	1	0	0	687
Petrusel; 2020	0	0	0	0	1	0	0	591
Farias; 2020	0	0	0	0	1	0	0	2,161
Dayem; 2021	0	0	0	0	1	0	0	5,320
Van Trant; 2021	0	0	0	0	1	0	0	384
Tan; 2023	0	0	0	0	1	0	0	289,356
Yuan; 2020	0	0	0	0	1	0	0	159,025
Park BK; 2022	0	0	0	0	1	0	0	7,445,944
Lee DY, 2018	0	0	0	0	1	0	0	4,945,886
Huang BZ, 2020	0	0	0	0	1	0	0	1,499,627
Er KC, 2016	0	0	0	0	1	0	0	699,115
White MJ, 2023	0	0	0	0	1	0	0	2,561,684
Setiawan VW, 2018	0	0	0	0	1	0	0	48,995
Safadi H, 2024	0	0	0	0	1	0	0	3,681,774
de Jong RGPJ, 2017	0	0	0	0	1	0	0	34,038
Jacobson; 2021	0	0	0	0	0	1	0	899
Rentsch; 2020	0	0	0	0	0	1	0	378,253
Koo; 2019	0	0	0	0	0	1	0	5,392,684

Keum; 2017	0	0	0	0	0	1	0	319
Goto; 2016	0	0	0	0	0	1	0	16,347
McCullough; 2018	0	0	0	0	0	0	1	138,266
Navarrete-Muñoz; 2016	0	0	0	0	0	0	1	477,199
Schernhammer; 2005	0	0	0	0	0	0	1	138,158
