# Supplementary material for: Metabolic Dysfunction and Related Dietary Exposures in Relation to Pancreatic Cancer Risk: A Review of the Evidence
Source: Int J Mol Sci. 2026 Aug 28;27(17):7707. doi: 10.3390/ijms27177707 (PMC13566534; doi:10.3390/ijms27177707)
Supplement: Supplementary file 1 [file ijms-27-07707-s001.zip › ijms-4396158-supplementaryS4.pdf]

Supplement S4. Summary of the evidence grading, key considerations, and rationale used to classify the strength of evidence for each metabolic and dietary exposure according to the WCRF Continuous Update Project (CUP) criteria.

Exposure	WCRF grade	Main justification
**Metabolic syndrome**	Probable	Consistent positive associations across multiple reviews, mainly prospective studies, biological plausibility
**Diabetes mellitus**	Probable	Large effect sizes, multiple cohort studies, consistent findings, possible reverse causality acknowledged
**Hyperglycaemia**	Probable	Strongest pooled RR, replicated across independent reviews, supported by Mendelian randomization evidence
**Abdominal obesity**	Probable	Consistent cohort evidence using WC and WHR
**BMI & Obesity**	Limited–suggestive	Small effect size and inconsistent findings across reviews
**Prediabetes**	Limited–suggestive	Positive association but based on few studies
**Low HDL**	Limited–suggestive	Positive but weak association and based on few studies
**Red and White Meat**	Limited–suggestive	Positive but weak association and based on few studies
**Fructose**	Limited–suggestive	Positive but weak association and based on few studies
**UPFs**	Limited–suggestive	Positive but weak association and based on few studies
**Hypertriglyceridaemia**	Limited–no conclusion	Conflicting results between reviews
**Protein intake**	Limited–no conclusion	Mostly null findings
**ASBs**	Limited–no conclusion	Multiple null findings
**Processed Meat**	Limited–no conclusion	Multiple null findings
**SSBs**	Limited–no conclusion	Null findings
**NSSBs**	Limited–no conclusion	Null findings
**GI/GL**	Limited–no conclusion	Null findings
